# Reactive stress-coping styles show more variable reproductive expenditure and fitness outcomes

**DOI:** 10.1038/s41598-020-66597-3

**Published:** 2020-06-12

**Authors:** Sean D. Twiss, Courtney R. Shuert, Naomi Brannan, Amanda M. Bishop, Patrick. P. Pomeroy

**Affiliations:** 1 Department of Biosciences, Durham University, Durham, DH1 3LE UK; 2 Alaska SeaLife Center, P.O. Box 1329, 301 Railway Ave, Seward, AK 99664 USA; 3 SMRU (Hong Kong), University of St Andrews, 1802 One Midtown, 11 Hoi Shing Road, Hong Kong Special Administrative Region, Hong Kong, China; 4 Sea Mammal Research Unit, University of St. Andrews, St. Andrews, KY16 8LB UK

**Keywords:** Behavioural methods, Behavioural ecology, Animal behaviour

## Abstract

Stress-coping styles dictate how individuals react to stimuli and can be measured by the integrative physiological parameter of resting heart-rate variability (HRV); low resting HRV indicating proactive coping styles, while high resting HRV typifies reactive individuals. Over 5 successive breeding seasons we measured resting HRV of 57 lactating grey seals. Mothers showed consistent individual differences in resting HRV across years. We asked whether proactive and reactive mothers differed in their patterns of maternal expenditure and short-term fitness outcomes within seasons, using maternal daily mass loss rate to indicate expenditure, and pup daily mass gain to indicate within season fitness outcomes. We found no difference in average rates of maternal daily mass loss or pup daily mass gain between proactive and reactive mothers. However, reactive mothers deviated more from the sample mean for maternal daily mass and pup daily mass gain than proactive mothers. Thus, while proactive mothers exhibit average expenditure strategies with average outcomes, expenditure varies much more among reactive mothers with more variable outcomes. Overall, however, mean fitness was equal across coping styles, providing a mechanism for maintaining coping style diversity within populations. Variability in reactive mothers’ expenditures and success is likely a product of their attempts to match phenotype to prevailing environmental conditions, achieved with varying degrees of success.

## Introduction

Despite the remarkable growth over recent decades in research on consistent individual differences in behaviour (under various terms such as personality, temperament, behavioural types and coping styles), there remains much debate about the adaptive value and fitness consequences of such inter-individual variation and the mechanisms by which such variation can be maintained within populations and species^1–6^. Ultimately the question remains; if one behavioural type has an apparent fitness advantage over others^7,8^, then how might variation in behavioural types be maintained in wild populations^4^? Smith and Blumstein’s^9^ meta-analysis showed that across a broad range of taxa, bolder individuals tended to achieve increased reproductive success, but often with greater risk of mortality, suggesting that balancing rewards and costs of boldness may vary in different contexts. Other studies have indicated that different behavioural types might prosper under different environmental conditions and that spatial or temporal variation in local conditions might maintain inter-individual variation within populations^10^. Therefore, the context- or situation-dependent balances between costs and benefits of different behavioural types have been considered key determinants of inter-individual variation within wild populations.

Most studies of personality are conducted in captive, laboratory settings, or at least temporarily remove animals from the wild in order to conduct behavioural tests in more controlled settings^10,11^ or monitor responses during capture and handling events^12,13^. As behavioural expression is often context dependent, patterns of inter-individual variation expressed in arguably abstract laboratory conditions or under acute stress may not always be expressed in the range of natural conditions a species experiences^14,15^, though see^16^. Thus, an increasing number of studies are attempting to assess personality and its consequences in natural wild settings, where inter-individual variation will have its ecologically and evolutionary relevant consequences^14,17–20^.

Consistent individual differences in behaviour are typically identified through repeatability of individual behaviour in experimental tests designed to yield metrics that can be interpreted in terms of a particular personality axis, such as the widely used bold-shy axis^7–11^ or variation in exploration^10^. While some behavioural test scenarios, such as an open field test, might be applicable to many species, tests often must be tailored to the particular mechanics (e.g. mode of locomotion) and sensory capabilities of the subject species. Thus, tests, and the behavioural metrics extracted, can be very species specific, particularly when attempting to conduct behavioural tests *in situ*, where local environmental conditions may also dictate experimental design^17,20,21^. Consequently, identifying clear commonality in the meaning of behavioural tests across multiple species can be challenging^22–24^. This limits the scope for comparative studies, which are key to unravelling broader evolutionary patterns and ecological relevance of consistent individual differences in behaviour^1,14,25–27^.

Physiological indicators of inter-individual differences can alleviate some of these limitations, especially if based on a fundamental physiological system that spans a wide taxonomic range. Resting heart rate variability (HRV) is a measure of the degree of variation in successive inter-beat (or R-R) intervals (IBI’s) while an individual is in a stationary condition and can be used to differentiate between individuals in terms of their stress-coping styles in a wide range of mammals^28–33^. The primary axis of coping styles is the pro-reactive axis. Proactive individuals form routines readily, express little behavioural flexibility and are less responsive to environmental stimuli. Conversely, reactive individuals are more flexible, making them more responsive to environmental stimuli^29,30,34^. HRV represents the combined effects of the parasympathetic and sympathetic branches of the autonomic nervous system on the heart’s pacemaker, the sinoatrial node^31,32^. At rest, reactive individuals have relatively high parasympathetic activity, resulting in higher HRV, whereas proactive individuals are dominated by sympathetic activity, resulting in reduced HRV^28,31,32^. However, there are very few studies that have measured a physiological indicator of individual coping style in a wild population^35^, with most physiological studies of coping style focusing on laboratory or captive animals^29,34^. Likewise, studies of resting HRV are based on data from laboratory bred specimens, domestic livestock or companion animals^28–33^.

With the development of heart rate monitors capable of recording IBI’s at millisecond precision it is possible to derive HRV measures on wild animals *in situ* while undergoing their normal daily activity. We deployed specially modified heart rate monitors on lactating grey seals (*Halichoerus grypus*) to assess across individual variation and within individual consistency in resting HRV over successive breeding seasons. Female grey seals have been shown to exhibit consistent individual differences in behaviour^17,36^ including in behavioural plasticity reflecting pro- and reactive stress-coping styles^17,37^. As coping styles represent a spectrum of approaches for dealing with life’s challenges^34,38,39^, it is likely that they are associated with inter-individual differences in reproductive success. Kontiainen *et al*.^40^ showed that proactive Ural owl (*Strix uralensis*) females produced more recruits, though such differential success again raises the question of how variation in coping styles is maintained. Monstrier *et al*.^12^ also showed enhanced offspring survival for proactive mothers in roe deer (*Capreolus capreolus*), but in this case success was habitat dependent, with reactive mothers faring better in certain habitats. Therefore, it is plausible that proactive and reactive grey seal mothers may vary in measures of reproductive success.

Grey seals in the UK are true capital breeders with spatially and temporally separate reproduction and foraging. Females fast during the autumnal breeding season, relying on stored reserves to provision themselves and their single pup throughout the 18–20 day lactation period, effectively providing a closed system for the assessment of reproductive expenditure^41^. All energy required for maintenance and reproduction is derived from foraging at sea outside of the breeding season. Thus, a female grey seal’s annual foraging success is expressed in her maternal post-partum mass, which sets limits on her consequent material expenditure on that year’s pup^41^. During lactation, maternal daily mass loss rate (kg/day) represents dynamic or average maternal expenditure during the current reproductive event, while pup daily mass gain rate (kg/day) provides an index of the short-term (within season) fitness outcomes of maternal expenditure, and the ratio of maternal daily mass loss rate to pup daily mass gain rate provides a measure of mass transfer efficiency^41^. This study system, therefore, provides the opportunity to examine how individual coping style is related to measures of individual condition, reproductive expenditure and success, allowing us to test for differences in fitness outcomes with respect to an individual’s position on the pro-reactive spectrum. Over 5 successive breeding seasons (2013–2017) we measured resting HRV of 57 lactating grey seals. First, we show that measures of resting HRV for individuals are repeatable over successive breeding seasons, indicating long-term consistent-individual differences in pro-reactivity. Then we ask whether proactive and reactive mothers differ in measures of maternal reproductive expenditure (maternal daily mass loss rate) and short-term (within breeding season) fitness outcomes (pup daily mass gain rate and mass transfer efficiency). However, as reactive individuals are predicted to exhibit greater flexibility than proactive individuals, either in terms of behaviour or physiology^29,30^, we predicted that reactive individuals would exhibit greater inter-individual variation relative to proactive individuals in these measures of maternal reproductive performance. Therefore, for each individual, we computed the modulus (the absolute value) of the deviance from the sample mean within each year for each of these metrics (maternal post-partum mass, maternal daily mass loss rate, pup daily mass gain rate and mass transfer efficiency) to determine whether reactive individuals tended to deviate more from annual means of these mass metrics compared to more proactive individuals.

## Results

### Repeatability of HRV across years

Repeatability of the resting HRV estimates for the 25 mothers that had heart rate recorded for more than one season was high (*R* = 0.630 ± 0.113, CI = 0.367–0.802, LRT p < 0.0001). Repeatability within individuals was also high in most individuals (*Ri* range = 0.302–0.999, median = 0.686, LQ = 0.582, UQ = 0.936).

### HRV and maternal allocation/pup growth

Resting HRV exhibited no discernible influence on the real values of maternal post-partum mass, maternal daily mass loss rate, pup daily mass gain rate, or mass transfer efficiency. Birth date was retained in the best model for absolute values of maternal post-partum mass, maternal daily mass loss rate, and pup daily mass gain rate (Table 1). Mothers that pupped later in the season tended to have lower post-partum masses, though post-partum mass was strongly influenced by individual ID (86% of variation accounted for by ID). As expected, post-partum mass was found to have a strong effect on both maternal daily mass loss rate and pup daily mass gain rate, where heavier mothers were found to have higher rates of mass loss, but also higher rates of pup mass gain (Table 2). However, for a given maternal post-partum mass, those that pupped later in the season tended to exhibit higher rates of maternal daily mass loss and pup daily mass gain (Table 2). Year also had a strong effect on the mass change parameters. In 2015 there were significantly higher rates overall for maternal daily mass loss (Kruskal-Wallis rank sum test by Year: Χ^2^ = 13.97, df = 4, p = 0.0074) and pup daily mass gain (Kruskal-Wallis rank sum test: Χ^2^ = 12.82, df = 4, p = 0.0123) relative to the other breeding seasons. However, the putative interaction between resting HRV and year was not retained in any of the confidence sets for any of the tested response variables. None of the parameters tested, however, appeared to explain observed differences in mass transfer efficiency as the null model was the only model remaining after model selection (Table 1). Device type (see methods) did not feature in any of the confidence sets for any of the tested response variables.

**Table 1:** Retained GLMMs for predicting mass and mass change proxies of short-term fitness, and the modulus of the deviance from the sample mean for each of these proxies.

Response variable	Model structure	df	AICc	ΔAIC	Weight
MPPM	Birthdate	4	210.2	0	0.287
	Null	3	215.1	4.89	0.025
MDML	Year + birth date + MPPM	9	238.5	0	0.262
	Year + MPPM	8	239.2	0.66	0.188
	Null	3	274.9	36.38	0
PDMG	Year + birth date + MPPM	9	252	0	0.418
	Year + MPPM	8	254.1	2.08	0.147
	Null	3	274.6	22.58	0
MTE	Null	3	277.6	0	0.425
MPPM DEVIANCE	Birth date + HRV	5	235.2	0	0.329
	Birth date	4	236.3	1.14	0.186
	Null	3	238.5	3.33	0.062
MDML DEVIANCE	Birth date + HRV	5	252.6	0	0.315
	Birth date	4	256.6	4.02	0.042
	Null	3	261.7	9.03	0.003
PDMG DEVIANCE	Birth date + HRV	5	265.5	0	0.389
	Birth date	4	268.2	2.65	0.103
	Null	3	274.2	8.67	0.005
MTE DEVIANCE	Null	3	271.4	0	0.224

Null model results are also provided for comparison, even if not retained in confidence set. All models contained ID as a random effect. N_obs_ = 95, N_ID_ = 57. Abbreviations: HRV = Resting heart rate variability, MPPM = maternal post-partum mass, MDML = maternal daily mass loss rate, PDMG = pup daily mass gain rate, MTE = mass transfer efficiency.

**Table 2:** Coefficient estimates for the retained fixed effects in the best model from Table 1 (ΔAIC = 0; Table 1) for each response variable.

Response variable	Fixed/random effect	Coefficient estimate	Standard error	P value	R^2^	CI
MPPM (86%)	Intercept	−0.047	0.135	**<0.0001**	0.092	0.013–0.225
	Birth date	−0.253	0.092	**0.006**	0.092	0.013–0.225
MDML (16%)	Intercept	−0.041	0.239	**<0.0001**	0.436	0.321–0.580
	Year (2014)	0.265	0.314	**0.0002**	0.007	0.000–0.080
	Year (2015)	0.944	0.273		0.105	0.019–0.242
	Year (2016)	0.304	0.284		0.011	0.000–0.092
	Year (2017)	0.141	0.277		0.003	0.000–0.064
	Birth date	0.165	0.093	0.076	0.039	0.000–0.148
	MPPM	0.571	0.090	**<0.0001**	0.347	0.208–0.489
PDMG (12%)	Intercept	−0.327	0.258	**<0.0001**	0.329	0.218–0.493
	Year (2014)	0.475	0.343	**0.0002**	0.020	0.000–0.111
	Year (2015)	0.810	0.298		0.071	0.005–0.196
	Year (2016)	0.262	0.310		0.007	0.000–0.081
	Year (2017)	−0.185	0.303		0.004	0.000–0.069
	Birth date	0.206	0.096	**0.031**	0.054	0.002–0.171
	MPPM	0.446	0.093	**<0.0001**	0.220	0.095–0.370
MPPM DEVIANCE (63%)	Intercept	0.063	0.117	**<0.0001**	0.126	0.036–0.274
	Birth date	0.255	0.103	**0.013**	0.086	0.010–0.217
	HRV	0.219	0.117	0.061	0.065	0.004–0.188
MDML DEVIANCE (37%)	Intercept	0.032	0.110	**<0.0001**	0.182	0.072–0.336
	Birth date	0.336	0.104	**0.0011**	0.129	0.031–0.270
	HRV	0.285	0.110	**0.0099**	0.095	0.014–0.229
PDMG DEVIANCE (7%)	Intercept	−0.003	0.099	**<0.0001**	0.149	0.050–0.300
	Birth date	0.334	0.098	**0.0007**	0.117	0.025–0.256
	HRV	0.235	0.010	**0.0186**	0.061	0.003–0.182

Mass-transfer efficiency (MTE) was best explained by the null model and therefore not included here. The percentage values beside each response variable denote the stochastic variation accounted for by ID derived from conditional and marginal coefficients of determination computed using r.squaredGLMM from the MuMIn package^93,96^. The table also provides *R*^2^ (with 95% confidence intervals) for fixed effects within the best models (derived using *r2beta*^96^). Abbreviations: HRV = Resting heart rate variability, MPPM = maternal post-partum mass, MDML = maternal daily mass loss rate, PDMG = pup daily mass gain rate, MTE = mass transfer efficiency.

### HRV and deviance from seasonal means

**Figure 1:**
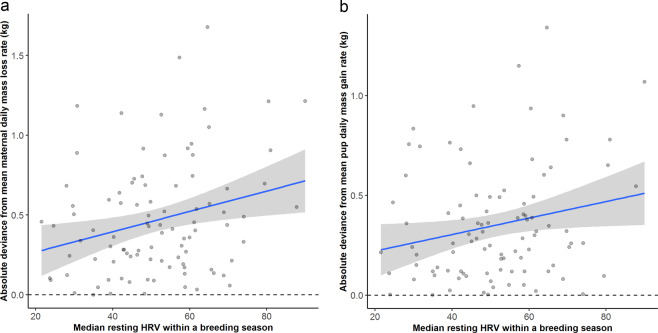
The effect of resting heart rate variability (HRV) on **(a)** the modulus of the deviance from annual mean values for rates of maternal daily mass loss, and **(b)** the modulus of the deviance from annual mean values for rates of pup daily mass gain. Annual mean represented by dashed line at y = 0.0. Line of best fit in blue with shaded area representing 95% CI. Points are raw data values.

Resting HRV appears to be influential in the degree of individuals’ deviation from annual mean rates of maternal mass loss and pup mass gain, though not for deviance in maternal post-partum mass or mass transfer efficiency. Females who gave birth later in the season were found to deviate more from the annual mean with respect to post-partum mass (Tables 1 and 2), which was again strongly influenced by individual ID (accounting for 63% of the model variation). While resting HRV was also retained in the best model, with more reactive individuals having more variable post-partum masses, the effect was non-significant. However, these results should be considered with caution as the null model was also retained in the confidence set (Table 1, ΔAIC Null = 3.33). By contrast, resting HRV was retained as a significant effect in our models of the degree of deviation from annual mean rates for maternal mass loss and pup mass gain (Fig. 1a,b). More reactive individuals appeared to exhibit greater variation in maternal daily mass loss rates as well as greater variation in pup daily mass gain rates than proactive individuals (Tables 1 and 2, Fig. 1a,b). Birth date was also retained, with mothers pupping later in the season exhibiting greater variation in rates of maternal mass loss and pup mass gain. In contrast to maternal post-partum mass, individual ID appeared to account for little variation in the deviance of maternal daily mass loss rate (37% of model variation) and pup daily mass gain rate (7% of model variation). Only the null model was retained in the confidence set for deviance in mass transfer efficiency model (Table 1). None of the confidence sets retained the putative interaction between Resting HRV and year and device type did not feature in any of the confidence sets for any of the tested response variables.

## Discussion

In our study we use a physiological measure of coping style, resting HRV, which is based on a physiological system that is highly conserved across vertebrates, and underpins behavioural patterns^29,30,34^. We show high inter-annual repeatability in resting HRV among female grey seals in an entirely natural setting, demonstrating across individual differences and long-term within individual consistency in stress-coping style on the pro-reactive spectrum. In addition, these different coping styles exhibit subtle differences in patterns of reproductive performance that suggest a mechanism by which inter-individual variation in coping style can be maintained within a wild population subject to natural environmental conditions.

Coping style did not influence real values of maternal size (post-partum mass), maternal performance (maternal daily mass loss rate, mass transfer efficiency), or short-term reproductive outcomes in terms of pup growth rates (pup daily mass gain rate). Therefore, across the sampled population, pro- and reactive mothers did not differ significantly in their initial state on arrival at the colony, their in-year expenditure, or their reproductive outcome. It is notable that we found no effect of coping style on the deviance from population mean values of maternal post-partum mass. Therefore, reactive mothers are not necessarily returning to the breeding colony in more variable states, suggesting that both pro- and reactive mothers are faring equally well overall in terms of net mass gain during the at-sea foraging phases of their lifecycle. It seems therefore that the mass change consequences of stress-coping style reveal themselves more on the breeding colony. Individual ID accounted for a large proportion of the variation in both absolute measures of maternal post-partum mass (86%) and deviance from mean post-partum mass (63%). This is indicative of individual differences in overall body size (as opposed to mass) and likely foraging success prior to the breeding season that are independent of coping style; a larger mother will always be larger. Whether pro- and reactive individuals adopt different, but equally successful, foraging strategies (e.g. specialist vs. generalist) while at sea is an area that requires further research.

By contrast, coping style appears to strongly influence the extent of inter-individual variability in our reproductive performance measures. Reactive mothers deviated more from the annual population mean in terms of their daily mass loss and in the daily mass gain of their pups. Proactive mothers exhibited a more consistent mean expenditure and outcome. This coping style effect was independent of pupping date and maternal post-partum masses which are known to influence maternal expenditure and consequent pup growth outcomes in grey seals^41^. These results demonstrate a reproductive pattern that can maintain inter-individual variation in stress-coping styles within a natural population. Overall, there was no net difference in fitness proxies across the spectrum of coping styles in terms of individual expenditure or outcomes. Therefore, there is likely no net selective advantage to being either proactive or reactive in terms of within season reproductive performance, at least under the environmental conditions that seals experienced during this study (indicated by the lack of evidence for an interaction between coping style and year in our models). There are very few empirical studies that directly show a link between coping style and fitness outcomes in natural environments, and the results of these efforts are mixed^12,40^. Kontiainen *et al*.^40^ found evidence that in conditions of temporally variable food availability proactive Ural owl mothers were more successful overall than reactive mothers. Monestier *et al*.’s^12^ study of coping-style in roe deer, showed that pro- and reactive mothers fare better in different habitats, providing a spatially dependent advantage to each coping style. Studies of personality (as opposed to coping style) in non-human animals suggest similar differential trade-offs for individuals across a personality spectrum. For example, the differential success of fast and slow exploring great tits (*Parus major*) across breeding seasons depending upon habitat resource quality^10^. A similar pattern of changing costs and benefits of boldness dependent upon spatio-temporal changes in resources was also found in Eurasian red squirrels *(Sciurus vulgaris*^42^). Such processes represent potential solutions to the conundrum of the maintenance of inter-individual variation in natural populations through differential costs and benefits dependent upon either spatial or temporal variation in environment^4^. In contrast, our results from studying female grey seals in their natural habitat suggested proactive mothers were not more successful, but instead were more uniform in their reproductive performance than reactive mothers, maintaining expenditure and outcomes closer to the mean values for the cohort.

Why should reactive mothers show greater variability in their reproductive expenditure (and consequently pup growth rates) than proactive mothers? Reactive individuals tend to be more responsive to environmental stimuli and consequently exhibit greater behavioural flexibility. Proactive individuals are less responsive to environmental stimuli, express little behavioural flexibility and form routines more readily^29,30,34,43^. Therefore, it is likely that reactive grey seal mothers tend to invest more or less than average in their current reproductive episode dependent upon the conditions prevailing at the time. A range of local, fine-scale spatial and temporal environmental factors have already been shown to strongly influence the behaviour of grey seal mothers on breeding colonies^44–50^. Grey seals are typically highly site-faithful to breeding colonies in the UK and tend to occupy similar parts of a colony year after year^51^. Furthermore, once mothers have pupped, they tend to be ‘tied’ to a small area of the colony due to the limited mobility of the pup^51^. However, conditions at each locality vary within and between breeding seasons^46–48^, and a grey seal is clearly unlikely to be able to predict future conditions prior to pupping. Therefore, we might expect reactive individuals to modify their behaviour and/or physiology to attempt to match the prevailing environmental conditions (phenotype-environment matching^4,43,52^), whereas proactive mothers will adopt an average ‘one size fits all’ approach. The reactive strategy is essentially a high reward but high-risk strategy. If a mother’s phenotype successfully matches to the prevailing conditions, then she is likely to be able to focus her investment in her current pup. If, however, she fails to appropriately match her phenotype to the conditions then she is likely to have to expend energy on other potentially costly activities (e.g. aggression, locomotion), reducing expenditure on her pup. There are various reasons why phenotypically plastic individuals may fail to successfully match their phenotype to the environment, including energetic and physical costs of, and limits to, plasticity but also where the environment changes too rapidly or unpredictably for an individual to make the appropriate changes in time^53^. Individuals with a reactive coping style may experience higher rates of mass loss due to the action of catabolic hormones (corticosteroids) which are in integral part of the suite of correlated traits defining coping styles^29,30,34,54^. Future studies could seek to measure not only coping style (using resting HRV), but also individual reactivity^30^ using stress hormones such as cortisol. Pro- and reactive individuals will likely respond differently to handling and sampling, which may provide a useful indicator of stress-reactivity if appropriately standardised.

Our results and their implications are particularly pertinent in the context of current rapid environmental change. Climate driven local weather patterns are becoming more variable and unpredictable^55^. Previous work has shown that grey seal behaviour and success on the breeding colony are weather dependent, particularly with respect to temperature and rainfall and their influence on thermoregulation and consequently behaviour^46–48,50^. However, grey seals are not alone in being subjected to ever more variable and unpredictable conditions during key phases of their lifecycle that are tied to specific seasons (e.g.^56^). Such changing environmental patterns will inevitably impact differentially upon proactive and reactive individuals within populations^27,57,58^. Therefore, assessing the extent of variation in coping styles within wild populations and their responses to changing environmental conditions is a vital step in understanding species resilience to rapid environmental change. Species able to cope with anthropogenic disturbance are likely to be those that contain some portion of behaviourally flexible individuals, rather than being species that are tolerant of human activities *per se*^59^. Although it remains unclear how directly linked the physiological underpinnings of coping styles and behavioural aspects of personality are^38,60^, coping styles are linked to the degree of behavioural flexibility that individuals may be able to express^29,30,34,38,39,43,61^. Given that the basic structure of the mammalian autonomic nervous system and the interplay of sympathetic and parasympathetic branches are highly conserved, it is probable that the behavioural and physiological distinctions between pro- and reactive types represent a fundamental biological pattern that can be observed in many mammalian species and indeed vertebrates more generally^29,30,34,43^. Consequently, findings from this study will have general applicability and broad relevance across vertebrate species.

## Methods

### Study site and years

The study was conducted during the annual grey seal breeding season at the Isle of May, Scotland (56.1856° N, 2.5575° W) in the years 2013 to 2017. From late October to early December, individual females spend 18–20 days on the island, during which time they each bear and nurse one pup, enter oestrus approximately 16 days post-parturition^62^, mate, and abruptly wean their pup around day 18 of lactation^41^. The Isle of May colony was part of a long-term study of grey seal reproductive energetics and behaviour by the Sea Mammal Research Unit, SMRU^41,63^. Known individuals can be identified using pre-existing brands, flipper tags and/or pelage patterns^41,64^. A subset of known breeding females are routinely handled each year by SMRU to obtain morphometric data, with individual females being captured and measured twice during their stay on the colony; once early in lactation (within 5 days of parturition) and once late in lactation (approx. day 14–16 after parturition). We used these handling events to deploy heart rate monitors on known females during the first capture, and to recover them at the second capture. Our focus was on re-deploying devices on the same individuals over multiple-years where possible to gain data on inter-annual consistency of resting HRV. Where individuals did not return or were not available to capture in a particular year, we then completed the annual sample size by including other mothers from the group of known individuals within the long-term study of grey seals on the Isle of May. Details of the standard capture procedure are published elsewhere^41,65,66^.

### Derivation of resting HRV estimates

**Figure 2:**
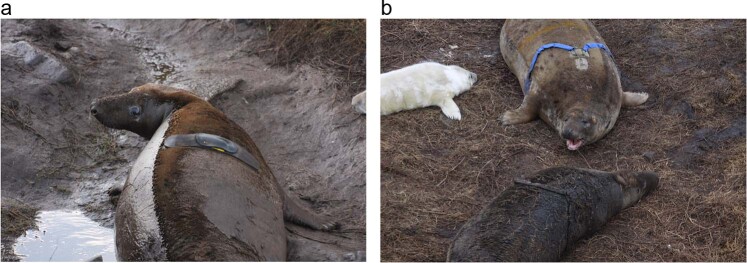
Two versions of telemetry devices were used to monitor heart rate variability for breeding female grey seals. (**a**) shows a neoprene strap containing a Polar H2/H3 monitor and Polar soft strap electrode, as used during the 2013 breeding season on the Isle of May, Scotland. (**b**) shows two neighbouring females equipped with Firstbeat heart rate monitors (as used in 2015–2017) with the extended electrode cables leading to silver chloride electrodes located immediately posterior of the left and right fore-flippers (as used in 2014–2017). The monitor is located centrally on the seals’ back. The monitor, cables and electrodes are protected by a covering of ballistic nylon. The female at the top of this image, is the same female as shown in Fig. 1a in the 2013 breeding season.

We instrumented focal seals with externally mounted heart rate monitors that provide millisecond precision measurements of IBIs through accurate detection of RR peaks^32,67^ and transmit these data to remote receivers. In the 2013 and 2014 seasons we used modified Polar H2/H3 monitors (Polar Electro Oy, Kempele, Finland) which transmitted IBI data to Polar RS800CX heart rate receivers. Polar devices have been validated for the recording of HRV in a range of domesticated mammalian species^31–33,67–71^. In 2013 the Polar monitors were attached to Polar soft strap electrodes extending 14 cm either side of the midline of the monitor. These electrode straps were mounted dorsally, just behind the right shoulder blade, extending laterally to the left and right. The entire unit was covered with a neoprene patch to provide protection and retain electrode gel (Fig. 2a; see^72^ for full design details). In 2014 this design was improved by replacement of the short Polar soft strap electrodes with two 50 cm protected cables leading to silver chloride electrodes located immediately posterior of the left and right fore-flippers providing more optimal electrode placement (Fig. 2b). The Polar system requires each monitor to be paired to a single receiver located within approximately 20 m^72^, which can constrain data collection especially in wild, free ranging animals. Therefore, in 2015–2017, we switched to using Firstbeat heart rate monitors (https://international-shop.firstbeat.com/product/team-pack/) as these provided a much increased signal transmission range (up to 200 m line of sight), and the capability to record multiple monitors (up to 80) at a single receiving station. These monitors were again modified with the extended electrodes (Fig. 2b). In all seasons, IBI data were collected during daylight hours only.

Artefacts in IBI data can lead to significant biases in estimates of HRV^32,67,73^. Sources of artefacts in IBI data can be intrinsic (e.g. arrhythmias, noise from muscle action potentials), or extrinsic (e.g. equipment malfunction, electromagnetic potentials), causing beats to be either missed or spuriously generated, leading to erroneously long or short IBI values^32,67,72^. We examined our raw IBI data for potential artefacts using Firstbeat Sports software (v.4.5.0.2^74^) and RHRV^75^ which detect extreme values and make corrections by deletion of spurious extra beats (extreme short IBIs) or interpolation for missing beats (extreme long IBIs). Artefacts that involve invariable sequences of IBIs (flats) or sequences of monotonically increasing or decreasing IBIs (stairs) cannot be corrected^33^, therefore these were identified using bespoke R scripts written by the authors (NB, AMB, SDT) to permit subsequent filtering of IBI traces with excessive flats and stairs^72^. IBI traces were then segmented into non-overlapping 300-second periods^32^ and HRV was computed as the root mean square of successive differences (RMSSD) between IBIs using RHRV. Although there are several measures of sympathovagal balance that can be computed from IBI data^32^, RMSSD is a preferred metric for use under free-running conditions because it is affected less by respiratory cycles^76,77^ and provides a measure that is relatively easy to interpret^32,77^. Any 300-second traces that had >5% flats or stairs were removed from subsequent analyses^32,33,72^.

Each IBI in the remaining traces was matched with a behavioural state derived from in-field video footage recorded concurrently with the IBI data collection. Video footage was decoded by experienced observers (n = 4) post-field season using a focal sampling protocol^78^ and a bespoke Visual Basic for Applications Macro in Microsoft Excel to record behaviours based on a pre-established ethogram^37,46,72,79^. In order to define resting HRV, the behavioural state of interest for this study was *Resting* (defined as the female remaining largely motionless, lying prone or supine with head in contact with the ground, eyes open or closed). All observers were trained to the same ethogram by SDT, and inter-observer reliability was tested on two hours of example video footage, showing high levels of agreement (ICC2 = 0.96, F_16,48_ = 109, p < 0.0001, 95% C.I. = 0.93–0.99; comparison across four independent observers based on 17 behavioural categories, of which Resting is one). Resting behaviour is relatively easy to identify reliably and tends to comprise the bulk of a breeding grey seal’s activity budget (typically over 60%^37,45,46,79–81^). Resting HRV was computed only from 300-second periods where the seal was at rest for ≥95% of the IBIs (and where the remaining time did not involve high energy behaviours such as locomotion or aggression). As our measure of resting HRV we computed the median RMSSD from all 300-second traces for each seal in each breeding season (number of 300 second traces per female per year ranged from three to 333, median = 32).

### Mass and mass change proxies of maternal expenditure and short-term fitness outcomes

Females and their pups were weighed at each capture as described in Pomeroy *et al*.^41^. Mass data were used to calculate three measures of maternal reproductive performance^41^; maternal post-partum mass (kg), maternal daily mass loss rate (kg/day), and pup daily mass gain rate (kg/day). Maternal post-partum mass was estimated using maternal daily mass loss rate to extrapolate a mother’s mass on the date of first capture to her parturition date as determined through in-field observations of the known females. Maternal post-partum mass provides a measure of maternal size and condition at a standardised time point (immediately after parturition) and is an index of realised somatic growth and foraging success prior to the breeding season^41^. As pup daily mass gain rate is influenced by pup physiology and behaviour as well as the maternal expenditure^41,65,80–84^ we also computed the ratio of daily rates of maternal mass loss: pup mass gain as a measure of maternal mass transfer efficiency.

We used our real values of maternal post-partum mass, maternal daily mass loss rate, pup daily mass gain rate, and mass transfer efficiency to examine whether proactive and reactive mothers differed in any of these proxies of maternal reproductive performance. However, as previous studies argue that reactive individuals are more likely to exhibit flexibility, either in terms of behaviour or physiology^29,30,34^, we also examined whether reactive individuals tended to deviate more from annual means of these mass metrics compared to more proactive individuals. Therefore, for every individual, we computed the modulus (the absolute value) of the deviance from the sample mean within each year for each of these metrics. We chose to use deviance from the annual mean (as opposed to a grand mean across all five years) to account for any interannual differences in seal behaviour and energetics which may have been driven by prevailing weather conditions at the breeding colony and consequent access to water across the population^37,46–50^.

### Statistical analysis

All analyses were conducted in R version 3.5.0^85^. For females with measures of resting HRV for more than one season (Table 3) we determined population level repeatability (*R*^86,87^) using the ‘rptR’ package^88^ with individual as a random effect and the number of measures per individuals as a fixed effect, with parametric bootstrapped 95% confidence intervals computed from 1,000 simulations. We also derived repeatability estimates for every individual (*Ri*) by dividing the between-individual variance (σ2α) by the sum of between-individual variance and the residual variance for each individual^20^.

**Table 3:** Summary of number of seals for which resting HRV estimates were obtained and the number of individuals with repeat measures across years.

No. of years with resting HRV estimate	No. of seals
1	32
2	16
3	6
4	2
5	1

We examined the effect of resting HRV on mass and mass change proxies of fitness by fitting generalized linear mixed-effects models (GLMM) within the R package lme4^89^. Female ID was included as a random effect to account for pseudoreplication of individuals across years^90,91^. We constructed separate models for each of the response variables; maternal post-partum mass, maternal daily mass loss rate, pup daily mass gain rate, and mass transfer efficiency, and for the deviance versions of these variables. In all models, response variables were standardised by z-transformation. The deviance measures were also log-transformed to meet heteroscedasticity assumptions and address overdispersion^92^. All response variables were checked for normality using Q-Q plots.

Our independent variables included our estimate of resting HRV for every individual. As individuals exhibited highly repeatable resting HRV across years, we used the median RMSSD across all years present for every individual as our estimate of resting HRV in these models. This was to avoid confounding the random effect of ID, included to account for pseudoreplication, with the highly individual measures of resting HRV. Global models for each response variable also included the date on which the female gave birth (birthdate, as number of days from 1^st^ January) as a covariate, with the sex of the pup, year and device type (Polar or Firstbeat) as factors. For all models (apart from those with maternal post-partum mass as the response variable), we included maternal post-partum mass as a covariate to account for the known positive relationship between maternal mass and daily rates of maternal mass loss and pup-growth^41^. All continuous covariates (resting HRV, birthdate and maternal post-partum mass) were standardised by z-transformation. Resting HRV and year were included in models as an interaction term to allow for an examination of whether different coping style fared better under different annual conditions^46–48,50^. For each mass response variable, the modelling procedure began by fitting the full (global) model using the Gaussian family and identity link. For model inference, we examined all plausible alternate models with reduced combinations of explanatory variables using the R function ‘dredge’ from the Package ‘MuMIn’^93^. The ‘best’ model was defined as the model with the lowest corrected Akaike’s information criterion (AICc). However, we also retained and examined all models within a confidence set, defined according to criteria established by Richards^94^. All models within a ∆AICc ≤ 6 of the ‘best’ model were retained within a preliminary confidence set. This initial confidence set was then subsetted, retaining only models with a ∆AICc value lower than more complex models within which they were nested. This approach avoids retaining overly complex models but also acknowledges that the model with the lowest AICc score is not necessarily the most parsimonious model^94,95^. For each response variable we also provide the output from the null model for comparison (models with no fixed effects and only the random effect).

We included no other interaction terms in the models reported here. However, where resting HRV and birthdate both featured in a confidence set, we did test alternative models with an interaction between resting HRV and birthdate, on the basis that seals with different coping styles might occupy the island at different stages of the breeding season. All these models performed worse (based on AICc) than the corresponding models lacking the interaction term, and therefore this interaction was not considered further. In all models the total number of observations was 95, and the total number of individuals was 57.

For each response variable we examined the relative contribution of ID as a random effect within the best model using *r.squaredGLMM* from the MuMIn package^93,96^ to provide conditional and marginal coefficients of determination. We also used *r2beta*^96^ to compute R^2^ (with 95% confidence intervals) for fixed effects within the best models.

### Ethics

All animal handling procedures conformed to the UK Animals (Scientific Procedures) Act, 1986 and were performed in collaboration with the Sea Mammal Research Unit (University of St. Andrews) operating under UK Home Office project licence #60/4009. The research presented here was approved by the Durham University Animal Welfare Ethical Review Board as well as by the University of St. Andrews Animal Welfare and Ethics Committee. Observational protocols were designed to conform to the ASAB/ABS guidelines for the treatment of animals in teaching research.

## Supplementary information

Supplementary Information.

## Data Availability

Data are available in the electronic supplementary material.
